# Neutrophil-to-lymphocyte ratio (NLR) predicts mortality in hospitalized geriatric patients independent of the admission diagnosis: a multicenter prospective cohort study

**DOI:** 10.1186/s12967-023-04717-z

**Published:** 2023-11-21

**Authors:** Mirko Di Rosa, Jacopo Sabbatinelli, Luca Soraci, Andrea Corsonello, Anna Rita Bonfigli, Antonio Cherubini, Riccardo Sarzani, Roberto Antonicelli, Giuseppe Pelliccioni, Roberta Galeazzi, Francesca Marchegiani, Salvatore Iuorio, Daniele Colombo, Maurizio Burattini, Fabrizia Lattanzio, Fabiola Olivieri

**Affiliations:** 1 Centre for Biostatistics and Applied Geriatric Clinical Epidemiology, IRCCS INRCA, Ancona, Italy; 2 Department of Clinical and Molecular Sciences, Università Politecnica Delle Marche, Ancona, Italy; 3 Laboratory Medicine Unit, Azienda Ospedaliero Universitaria Delle Marche, Ancona, Italy; 4 Unit of Geriatric Medicine, IRCSS INRCA, C.da Muoio Piccolo, 87100 Cosenza, Italy; 5 Department of Pharmacy, Health and Nutritional Sciences, University of Calabria, Rende, Italy; 6 Scientific Direction, IRCCS INRCA, Ancona, Italy; 7 Geriatria, Accettazione Geriatrica e Centro di ricerca per l’invecchiamento, IRCCS INRCA, Ancona, Italy; 8 Internal Medicine and Geriatrics, IRCCS INRCA, Ancona, Italy; 9 Cardiology Unit, IRCCS INRCA, Ancona, Italy; 10 Neurology Unit, IRCCS INRCA, Ancona, Italy; 11 Clinic of Laboratory and Precision Medicine, IRCCS INRCA, Ancona, Italy; 12 Department of Anaesthesiology, IRCSS INRCA, Ancona, Italy; 13 Respiratory Unit, IRCCS INRCA, Casatenovo, Italy; 14 Internal Medicine Department, IRCCS INRCA, Osimo, Italy

**Keywords:** NLR, Neutrophil-to-lymphocyte ratio, Older patients, Hospitalization, Mortality, Personalized medicine

## Abstract

**Background:**

The Neutrophil-to-lymphocyte ratio (NLR) is a marker of poor prognosis in hospitalized older patients with different diseases, but there is still no consensus on the optimal cut-off value to identify older patients at high-risk of in-hospital mortality. Therefore, in this study we aimed at both validating NLR as a predictor of death in older hospitalized patients and assess whether the presence of specific acute diseases can modify its predictive value.

**Methods:**

This prospective cohort study included 5034 hospitalizations of older patients admitted to acute care units in the context of the ReportAge study. NLR measured at admission was considered as the exposure variable, while in-hospital mortality was the outcome of the study. ROC curves with Youden’s method and restricted cubic splines were used to identify the optimal NLR cut-off of increased risk. Cox proportional hazard models, stratified analyses, and Kaplan–Meier survival curves were used to analyse the association between NLR and in-hospital mortality.

**Results:**

Both continuous and categorical NLR value (cut-off ≥ 7.95) predicted mortality in bivariate and multivariate prognostic models with a good predictive accuracy. The magnitude of this association was even higher in patients without sepsis, congestive heart failure, and pneumonia, and those with higher eGFR, albumin, and hemoglobin (*p* < *0.001).* A negative multiplicative interaction was found between NLR and eGFR < 45 (*p* = *0.001).*

**Conclusions:**

NLR at admission is a readily available and cost-effective biomarker that could improve identification of geriatric patients at high risk of death during hospital stay independent of admitting diagnosis, kidney function and hemoglobin levels.

**Supplementary Information:**

The online version contains supplementary material available at 10.1186/s12967-023-04717-z.

## Background

Medical progress in the management of acute diseases has reduced overall hospital death rate, that however, is high in geriatric patients. Predictors of prognosis for use in routine clinical practice are an urgent need for geriatric hospitalized patients. Since neutrophils and lymphocytes represent the larger percentage of all the immune cells circulating in the bloodstream, the neutrophil/lymphocyte ratio (NLR) is a simple index of inflammation. NLR is characterized by a progressive increase in the first years of life, and then remains at similar levels during healthy aging. NLR increased values are associated with lifestyle and environmental stressors impacting on immune system [1]. Increasing evidence strongly support the association between increased NLR values and a variety of pathological conditions characterized by a proinflammatory status. NLR increased values were observed in infectious diseases, such as acute exacerbations of chronic obstructive pulmonary disease [2], pneumonia [3], COVID-19 [4, 5] and sepsis [6], and non-infectious diseases, including atherosclerotic coronary artery disease (CAD) [7, 8], stroke [9], neurodegenerative diseases [10], cancers [11, 12], kidney diseases [13], and acute kidney injury (AKI) [14]. In all these conditions, NLR is emerging as a biomarker of disease severity and for mortality risk estimation. While a large body of literature highlighted the prognostic potential of the NLR, the translation of this promising marker into the clinical setting remains highly challenging as the strength of association between NLR and overall survival varies between published studies. Although there are still no consensually accepted normal values for this parameter, there is a proposal to consider an interval of 1–2 as a normal value, an interval of 2–3 as a grey area indicating subclinical inflammation and values above 3 as inflammation [15]. NLR measurement is cheap and easily available through patients’ baseline and routine investigation of complete blood count [16]. NLR can be a universally available and inexpensive prognostic marker. Despite evidence of strong prognostic potential, no conclusive results were provided on the optimal cut-off value defining high-risk NLR. Furthermore, limited understanding of the strength of association between NLR and clinical outcomes in specific patient subgroups are limiting the adoption of the NLR as a tool for prognosis or clinical decision making. Identifying patients for whom the NLR harbors maximum prognostic potential and suggesting corresponding group-specific cut-offs for high NLR, is a challenge. However, geriatric patients are characterized by conditions such as multi-morbidity and frailty [17, 18], and hospitalized geriatric patients are extremely heterogenous. In this work, we aimed to a) verify NLR relevance as prognostic indicator of short-term mortality in geriatric patients at the time of hospital admission; b) investigate the effect modification of admission diagnosis and laboratory abnormalities on the association between NLR and mortality. The present study in a large prospective cohort of hospitalized older patients may facilitate the prospective clinical validation of the NLR as a tool for mortality risk stratification in geriatric hospitalized patients and assess its prognostic importance independent of admission diagnosis.

## Materials and methods

### Study population

In this study, we used data derived from the Report-AGE project, a large-scale observational study on the health conditions of older adults’ patients (over 65 years) admitted to the acute care wards of the Italian National Institute of Health and Science on Ageing (IRCCS INRCA) [19] (Trial Registration no. NCT01397682).

Briefly, all patients consecutively admitted to participating wards from September 2011 to October 2021 were asked to participate. After obtaining written informed consent, all patients underwent comprehensive geriatric assessment (CGA) by Inter-RAI Minimum Data Set acute care (MDS-AC) [20] conducted both at the time of hospitalization and at discharge. Criteria for inclusion were age ≥ 65 years, length of stay more than 24 h, and signed informed consent. Only patients affected by hematological cancers and COVID-19 were excluded. Routine laboratory parameters were available for 5, hospitalizations (4020 subjects and 1014 repeated hospitalizations). Participating physicians and nurses were specifically trained before starting recruitment, as previously described [17, 21]. Data on disease history of all patients recruited for this study were obtained from the medical records. The main acute and chronic diseases at hospital admission were coded in accordance with the International Classification of Diseases, 9th revision (http://www.icd9data.com/). Multimorbidity was measured by Charlson Comorbidity Index [22].

### Laboratory parameters

Laboratory parameters measured at hospital admission included blood cell counts, creatinine, albumin, hemoglobin, and aspartate aminotransferase/alanine aminotransferase (AST/ALT), and were assessed with standardized procedures. eGFR was calculated by using BIS Equation [23]:

$${\text{BIS1 eGFR}}\, = \,{3},{736}\, \times \,{\text{S}}_{{{\text{Cr}}}}^{{{-}0.{87}}} \, \times \,{\text{age}}^{{{-}0.{95}}} [ \times \,0.{\text{82 if women}}]$$

where S_Cr=_serum creatinine.

Hypoalbuminemia was defined as having serum albumin levels < 3.5 g/dL.

### Outcome

Outcome measure of interest was in-hospital death. Patients were followed from hospital admission until died in hospital or discharged alive. Length of hospital stay was calculated as the time from the patient’s admission to the acute care unit until discharged or died in the hospital.

### Statistical analysis

Means with standard deviations were used to describe continuous variables with normal distribution (assessed with Shapiro–Wilk test); absolute frequencies and percentages were used for categorical variables.

The Chi-square test and the Student’s *t* test were performed to compare variables between groups (survived vs deceased patients), as appropriate. One-way ANOVA with post-hoc Bonferroni test for multiple comparisons were used to evaluate differences in mean NLR levels between patients with distinct diseases at hospital admission. In order to identify the NLR cut-off of increased risk in our population we followed a two-step approach; first, we applied the Receiver operating characteristics (ROC) curve analysis with Youden’s method to maximize the sum of sensitivity and specificity; second, to examine the possibility of non-linear associations between NLR and study outcomes, we used restricted cubic splines with best knots placed at 25^th^, 50^th^, and 75^th^ percentile of NLR distribution. NLR was then dichotomized according to the identified cut-off and its association with in-hospital mortality was inspected by using Kaplan-Meyer curves and cox regression analyses. To account for sex differences in identified cut-offs, we also performed weighted ROC curve analysis and sex-specific restricted cubic splines. The association of both continuous and categorical NLR with in-hospital mortality was then assessed by using bivariate and multivariate Cox regression models; Model 1 was adjusted for age and sex; Model 2 was adjusted for age, sex, main diagnoses at hospital admission and Charlson index; Model 3 was adjusted for age, sex, eGFR, albumin, hemoglobin, and AST/ALT; Model 4 was adjusted for age, sex, main diagnoses, Charlson index, eGFR, albumin, hemoglobin, AST/ALT. We finally examined the possible effect modification in the baseline categorical NLR-mortality association across strata of clinical and laboratory values.

All the analyses were performed with SPSS 25.0 (IBM, Chicago, IL, USA), Stata 15.1 (StataCorp LP, College Station, TX, USA), and R 4.6. *P* values less than 0.05 (two-sided) were considered statistically significant.

## Results

Demographic, clinical and laboratory characteristics of hospitalizations included in the present analysis are reported in Additional file 1. Overall, the 5,034 patients included in the study had a mean age of 86.6 ± 6.4 years, were more commonly women (57.5%), with a Charlson index ≥ 2 (55.2%), and a mean length of stay of more than 9 days. During the hospital stay, 611 out of 5,034 hospitalizations led to death (12.1%). Patients who died were significantly older, had a higher prevalence of several acute diseases, including sepsis, lung infections, cardiac arrhythmias and heart failure, as well as a lower glomerular filtration rate compared to those who survived. As regards the laboratory values, mean levels of NLR, AST, ALT, white blood cells (WBC), and creatinine were significantly higher in deceased patients; conversely, albumin and hemoglobin levels were significantly lower. As regards the NLR distribution, median [interquartile range, IQR] NLR values were 5.9 [3.3–11.4] and were also significantly higher in deceased patients (11.0 [6.0–22.0]) compared to survived ones (5.5 [3.1–10.3]).

NLR mean values were explored in the overall population and in patients with specific diseases at hospital admission (Table 1). Patients with sepsis and lung infections had the highest NLR values, followed by those with metabolism disorders and diabetes, heart diseases, and gastrointestinal pathologies; similar results were shown in analyses reporting median and IQR instead of mean (SD) values (Additional file 2); one-way ANOVA with Bonferroni post-hoc test showed significant differences in NLR mean levels depending on the main cause for hospital admission (Additional file 3). The most significant differences were observed between patients with sepsis, pneumonia, and cerebrovascular diseases compared to those affected by other diagnoses.

**Table 1:** NLR (mean and standard deviation) for the main diagnosis at hospital admission

NLR by Main diagnosis	Mean ± sd	Overall p
		< 0.001
1. Diabetes	9.35 ± 8.04	
2. Metabolism and nutrition disorders	10.22 ± 11.25	
3. Delirium and other psychiatric disorders	8.45 ± 8.05	
4. Cerebrovascular disease	4.71 ± 4.81	
5. Cancer	6.28 ± 5.78	
6. Anemia	8.27 ± 8.94	
7. Dementia or other disorders of the nervous system	5.27 ± 6.35	
8. Heart failure and heart disease	10.06 ± 8.90	
9. Hypertension or cardiac arrhythmias	8.72 ± 7.28	
10. Lung infections	12.51 ± 12.82	
11. Gastrointestinal pathologies	9.99 ± 11.23	
12. Genitourinary pathologies	10.00 ± 10.29	
13. Sepsis	14.82 ± 15.25	
14. Other	9.65 ± 10.28	

**Fig. 1:**
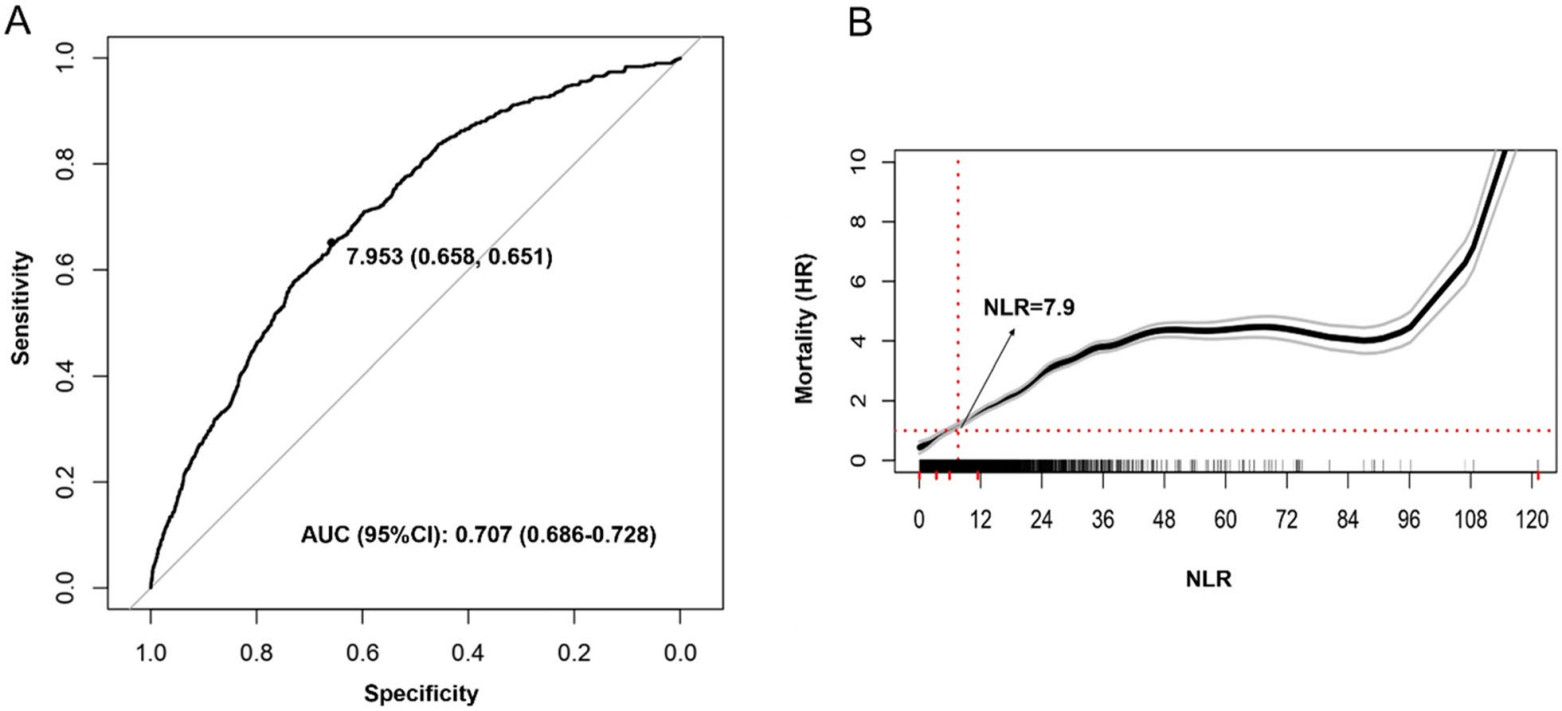
Selection of the optimal cut-off of NLR for increased risk of in-hospital mortality. Selection of the optimal cut-off of NLR by using ROC curve analysis with Youden’s method (**A**) and restricted cubic spline with 3 knots (**B**). Red ticks on x-axis represent quartiles of NLR distribution

Cut-off points of NLR for increased risk of in-hospital death were substantially coincident when performing ROC analysis with Youden’s method and spline regression models (Fig. 1). Indeed, ROC analysis identified in 7.95 the NLR cut-off value able to efficiently capture the patient’s risk of death with a sensitivity of 66% and specificity of 65%; the predictive accuracy was good (AUC = 0.707; 95%CI 0.686–0.728). The analysis of the association between NLR and death through restricted cubic splines highlighted the non-linear dose–response relationship; in patients with NLR above the threshold of 7.95, the risk of death increased progressively but becomes quasi-exponential in NLR range between 7.95 and 35. Similar findings were shown in sex-stratified analyses (Additional file 5: Fig. S1a, Additional file 6: Fig. S1b, and Additional file 7: Fig. S1c).

**Fig. 2:**
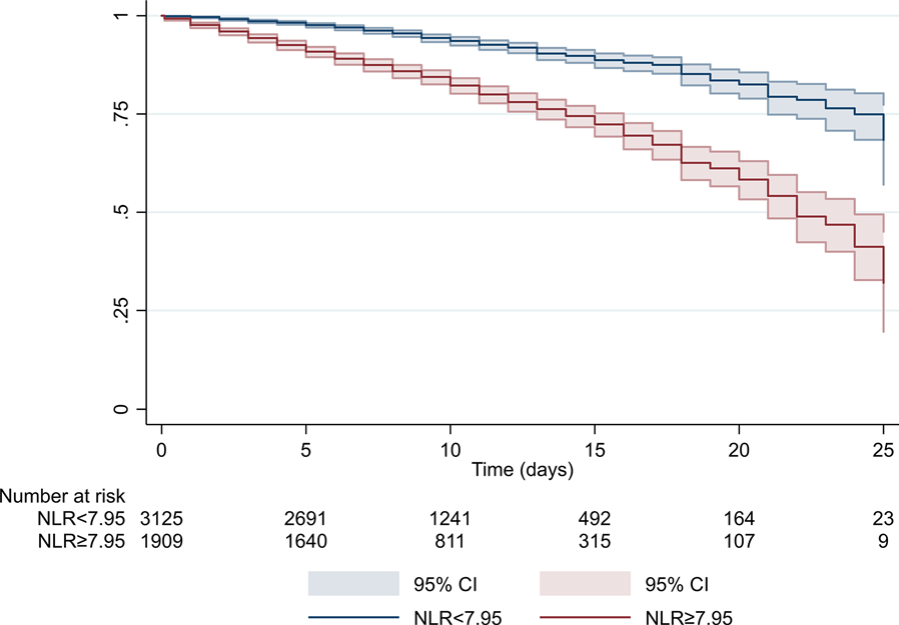
Kaplan Meier survival estimates for dichotomized NLR in the overall study population

Survival analysis using Kaplan Meier curves showed that patients with NLR ≥ 7.95 had an increased risk of in-hospital death compared to those with lower NLR levels (Fig. 2, log rank *p* < *0.001*). Similar findings were reported after stratifying by sex of included patients (Additional file 8: Fig. S2a and Additional file 9: Fig. S2b).

Cox regression analysis showed that continuous NLR was significantly associated with in-hospital mortality in both bivariate and all multivariate models; furthermore, after categorization, NLR ≥ 7.95 was shown to be significantly associated with study outcomes in all regression models (Table 2).

**Table 2:** Cox regression analysis showing the bivariate and multivariate association between NLR and death

	Continuous NLR	NLR ≥ 7.95
	HR (95% CI)	HR (95% CI)
Model 1	1.028 (1.024–1.032)	2.773 (2.345–3.279)
Model 2	1.025 (1.021–1.030)	2.487 (2.099–2.948)
Model 3	1.020 (1.015–1.025)	1.990 (1.664–2.380)
Model 4	1.019 (1.013–1.024)	1.862 (1.549–2.239)

Model 1: age & sex-adjusted

Model 2: model 1 adjusted for the main diagnoses, and Charlson index

Model 3: model 1 adjusted for eGFR, albumin, hemoglobin, and AST/ALT

Model 4: model 2 + model 3

To assess the effect modification of distinct diseases and laboratory values on the association between NLR and study outcome, we performed stratified cox regression models reported in Additional file 4. Association between NLR (both continuous and categorical) and death was found to be consistent across all diseases and lab abnormalities, but, interestingly, predictive accuracy was found to be higher and slightly more pronounced in patients without high-risk acute conditions (e.g. sepsis, pneumonia, and congestive heart failure), and among those with more preserved kidney function, normal albumin levels and normal hemoglobin (Additional file 4). Also, a multiplicative age and sex-adjusted negative interaction was found between continuous NLR and eGFR on mortality risk (*p* = *0.041)* and between categorical NLR and eGFR < 45 ml/min/1.73 m^2^ (*p* < *0.001*). Indeed, despite NLR was shown to progressively increase with eGFR decline and both variables predict mortality in fully adjusted models, they negatively interacted in predicting death. Furthermore, NLR/BIS ratio was associated with death with a higher strength in patients with eGFR ≥ 45 (fully adjusted HR: 4.80, 2.61–8.83) compared to eGFR < 45 (fully adjusted HR: 1.55, 1.36–1.77, *p* < *0.001*). These results were also confirmed in fully-adjusted models containing a 4-level analytical variable including categories of NLR (< and ≥ 7.95) and eGFR (< and ≥ 45 ml/min/1.73 m^2^). Indeed, compared with patients with eGFR < 45 and NLR < 7.95, those with eGFR ≥ 45 and NLR ≥ 7.95 were characterized by an increased risk of death in fully adjusted models A and B, and in C and D fully adjusted models not containing albumin (*p* < *0.001*).

## Discussion

In this retrospective study of 5,034 consecutive older patients, we investigated the association between NLR—assessed at admission—and in-hospital mortality. We showed that patients deceased during the hospital stay had a significantly higher NLR compared to those who survived. Furthermore, despite several acute conditions significantly contributed to patient’s hospital admission and prognosis, NLR predictive ability was independent from the admission diagnosis and was even better in patients without critical illnesses and in those with preserved hemoglobin and renal function. Finally, a negative multiplicative negative interaction was found between NLR and GFR. To the best of our knowledge, this is the first study assessing the predictive value of NLR in hospitalized geriatric patients independently of the admission diagnosis and across a large spectrum of disease states and laboratory parameters. Analysis of the NLR values according to the main diagnoses revealed significant differences among conditions, with sepsis and pneumonia displaying the highest values Despite the high heterogeneity observed according to the main diagnosis, we were able to obtain a single NLR cutoff of 7.95, which was able to predict in-hospital mortality independent of the admission diagnosis, age, sex, comorbidities, and other biochemical confounders. Previous studies evaluated the prognostic value of NLR in hospitalized patients, mostly in specific diseases or conditions [24–26]. While a substantial agreement has been reached on the association between high NLR and poor outcomes, a high heterogeneity of the proposed cutoffs has been highlighted [24, 27–29]. In our population, we observed a relatively high mean NLR of 9.5, which could be related to pre-existing comorbidities, most of which are accompanied by the chronic low-grade proinflammatory status that characterizes age-related diseases [30]. The detrimental role of heightened inflammation and depressed adaptive immunity is evident not only in infectious diseases, where most of the studies on NLR have focused, but also in other non-communicable disorders, where the persistence of chronic inflammation could accelerate organ damage (e.g. type 2 diabetes), prevent functional recovery (e.g. major cardiovascular events), or promote cancer progression. In this framework, NLR could be considered as a proxy measure of the imbalance between innate and acquired immunity which has been identified as a major feature of immunosenescence and determinant of inflammaging [31].

Albeit we showed that a single NLR cutoff applies to a highly heterogeneous population of older patients, we recognize the importance of interpreting NLR values also as a continuous variable. Indeed, we reported a linear relationship between NLR values < 40 and mortality risk, which was further confirmed by the multivariable analysis, that resulted in an additional 2% mortality risk for each 1-point increase of NLR. These results suggest that NLR provides an easy-to-interpret outcome predictor but that specific cutoff values should be computed according to different clinical settings.

In a secondary analysis, we performed multiple stratifications according to the presence of specific diagnoses and the levels of laboratory variables that were used to infer the general condition of the patients at the time of admission, such as eGFR, albumin, and hemoglobin, which are associated with mortality [32, 33]. The survival analysis revealed that the discriminatory ability of NLR was consistently higher in those patients without diseases characterized by the highest mortality rates and NLR values, including sepsis, pneumonia, heart failure, and with absence of either hypoalbuminemia, impaired renal function, or anemia. The observed results could be explained by the wider distribution of NLR in patients without the conditions associated with the highest NLR values, which invariably increases its ability of effectively discriminate patients according to their survival status. While increasing attention has been devoted to NLR in patients with acute conditions, such as sepsis, myocardial infarction, and COVID-19, our results remark on the importance of assessing this parameter also in non-critical settings. This is further confirmed by the negative interaction found between NLR and eGFR; indeed, whereas both increasing NLR and decreasing eGFR were significantly associated with mortality in fully-adjusted models, their prognostic interaction was negative and multiplicative in both categorical and continuous models. More in detail, patients with eGFR ≥ 45 ml/min/1.73 m^2^ and high NLR shared a significant higher risk of death compared to those with eGFR < 45 and normal NLR, thus underlining the importance of considering NLR in addition to eGFR in stratifying the prognosis of older patients in the acute care setting, where the evaluation of eGFR only could be biased by rapid changes of serum creatinine during hospital stay [34, 35].

The major strengths of our study are the large sample size, the specific focus on a population of older subjects (age > 80 yrs.), and the inclusion of all patients admitted to hospital during the observation period, which minimized the risk of selection bias. Moreover, we provided evidence that a variable obtained at no additional cost could improve the predictive ability of the other routinely assessed biomarkers, including eGFR.

Some limitations have to be highlighted. First, our analysis relied on a single blood cell count, which did not allow us to explore potential relationships between fluctuations of NLR during hospital stay and study outcome. In this regard, repeated measurements of NLR during the in-hospital stay may provide additional prognostic information. Second, diagnosis of diseases was retrospectively derived from ICD-9 codes, with potential underestimation of disease prevalence at hospital admission; third, we could not include sensitive measures of disease severity which may improve prognostic risk stratification in hospitalized older patients. However, our study has also several strengths, such as the large sample size, the prospective study design, the comprehensive characterization of hospitalized older patients, and the use of multiple statistical approaches to test the predictive accuracy of NLR across a wide range of disease conditions.

## Conclusions

In our study, we showed that a readily available and cost-effective biomarker such as NLR could provide additional guidance in identifying geriatric patients at higher risk of death during their in-hospital stay already at the time of admission. As a future development, other indices derived from the complete blood count, as well as the study of the additional parameters provided by most hematology analyzer manufacturers reflecting immune cell differentiation and activation, could expand, with limited efforts, the set of predictive biomarkers available for risk stratification.

### Supplementary Information

**Additional file 1: **Baseline characteristics of the study population in general and after stratifying by in-hospital status.

**Additional file 2: **NLR (median and interquartile range) for the main diagnosis at hospital admission.

**Additional file 3: **Comparisons of mean NLR values by main diagnosis at hospital admission (one-way ANOVA and post-Hoc Bonferroni test).

**Additional file 4: **Assessment of the effect modification of diseases and lab values on the association between NLR (both continuous and categorical) and death.

**Additional file 5: Figure S1a.** Optimal NLR cut-off for increased risk of in-hospital mortality in men and women. Selection of the optimal cut-off of NLR for increased risk of in-hospital mortality by using sex-weighted and age-adjusted ROC curve in the study population.

**Additional file 6: Figure S1b.** Restricted cubic spline showing the non-linear relationship between NLR and in-hospital mortality among male patients. Red ticks on x-axis represent quartiles of NLR distribution.

**Additional file 7: Figure S1c.** Restricted cubic spline showing the non-linear relationship between NLR and in-hospital mortality among female patients. Red ticks on x-axis represent quartiles of NLR distribution.

**Additional file 8: Figure s2a.** Kaplan Meier curves showing survival of male patients according to NLR categories.

**Additional file 9: Figure s2b.** Kaplan Meier curves showing survival of female patients according to NLR categories.

## Data Availability

Anonymised data and code used in conducting the analyses will be made available upon request directed to the corresponding author.

## Abbreviations

AKI: Acute kidney injury
ALT: Alanine aminotransferase
AST: Aspartate aminotransferase
CAD: Coronary artery disease
CGA: Comprehensive geriatric assessment
CI: Confidence interval
eGFR: Estimated glomerular filtration rate
IQR: Interquartile range
HR: Hazard ratio
MDS-AC: Minimum data set acute care
NLR: Neutrophil-to-lymphocyte ratio
ROC: Receiver operating characteristic
WBC: White blood cells
