# Supplementary material for: Neutrophil-to-lymphocyte ratio (NLR) predicts mortality in hospitalized geriatric patients independent of the admission diagnosis: a multicenter prospective cohort study
Source: J Transl Med. 2023 Nov 21;21:835. doi: 10.1186/s12967-023-04717-z (PMC10664513; doi:10.1186/s12967-023-04717-z)
Supplement: Supplementary file 1 — Additional file 1: Baseline characteristics of the study population in general and after stratifying by in-hospital status. [file 12967_2023_4717_MOESM1_ESM.docx]

**Additional File 1: Baseline characteristics of the study population in general and after stratifying by in-hospital status.**

|  | | **Total** | **Survived** | **Deceased** | **p** |
| --- | --- | --- | --- | --- | --- |
|  | | **n=5,034** | **n=4,423** | **n=611** |  |
| Age, mean±sd | | 86.6±6.4 | 86.2±6.5 | 89.1±5.4 | <0.001 |
| Female sex, n(%) | | 2,893(57.5%) | 2,535(57.3%) | 358(58.6%) | 0.549 |
| Length of stay (days), mean±sd | | 9.4±5.1 | 9.6±5.0 | 8.3±5.9 | <0.001 |
| NLR, mean±sd | | 9.4±10.5 | 8.5±9.1 | 16.1±16.1 | <0.001 |
| Neutrophils (%), mean±sd | | 77.0±12.4 | 76.0±12.4 | 84.4±10.2 | <0.001 |
| Lymphocytes (%), mean±sd | | 15.2±10.0 | 15.9±10.1 | 9.9±7.8 | <0.001 |
| Neutrophils (*10^3^/mm^3^), mean±sd | | 8.3±6.1 | 7.8±5.2 | 11.6±9.8 | <0.001 |
| Lymphocytes (*10^3^/mm^3^), mean±sd | | 1.4±3.2 | 1.4±2.8 | 1.3±5.2 | 0.724 |
| Creatinine, mg/dL, mean±sd | | 1.5±1.2 | 1.4±1.1 | 1.9±1.4 | <0.001 |
| BIS1 eGFR, ml/min/1.73 m^2^, mean±sd | | 46.4±22.1 | 47.5±21.3 | 38.9±25.7 | <0.001 |
| Hemoglobin, g/dL, mean±sd | | 11.3±2.0 | 11.3±2.0 | 10.8±2.0 | <0.001 |
| White blood cells (*10^3^/mm^3^), mean±sd | | 10.4±7.3 | 9.9±6.0 | 13.6±12.8 | <0.001 |
| Albumin, g/dL, mean±sd | | 3.2±0.6 | 3.3±0.6 | 2.8±0.6 | <0.001 |
| AST, U/L, mean±sd | | 42.8±170.3 | 38.7±103.5 | 72.5±401.3 | <0.001 |
| ALT, U/L, mean±sd | | 35.9±124.5 | 33.6±88.4 | 53.2±270.3 | 0.001 |
| AST/ALT, mean±sd | | 1.4±0.6 | 1.3±0.5 | 1.5±0.9 | <0.001 |
| Main diagnosis at admission, n(%) | |  |  |  | <0.001 |
|  | Diabetes | 57(1.1%) | 50(1.1%) | 7(1.2%) |  |
|  | Metabolism and nutrition disorders | 369(7.3%) | 299(6.8%) | 70(11.5%) |  |
|  | Delirium and other psychiatric disorders | 69(1.4%) | 66(1.5%) | 3(0.5%) |  |
|  | Cerebrovascular disease | 259(5.2%) | 250(5.7%) | 9(1.5%) |  |
|  | Cancer | 188(3.7%) | 162(3.7%) | 26(4.3%) |  |
|  | Anemia | 410(8.1%) | 388(8.8%) | 22(3.6%) |  |
|  | Dementia or other disorders of the nervous system | 528(10.5%) | 514(11.6%) | 14(2.3%) |  |
|  | Heart failure and heart disease | 151(3.0%) | 117(2.7%) | 34(5.6%) |  |
|  | Hypertension or cardiac arrhythmias | 289(5.7%) | 228(5.2%) | 61(10.0%) |  |
|  | Lung infections | 787(15.6%) | 661(14.9%) | 126(20.6%) |  |
|  | Gastrointestinal pathologies | 519(10.3%) | 487(11.0%) | 32(5.2%) |  |
|  | Genitourinary pathologies | 510(10.1%) | 452(10.2%) | 58(9.5%) |  |
|  | Sepsis | 235(4.7%) | 169(3.8%) | 66(10.8%) |  |
|  | Other | 663(13.2%) | 580(13.1%) | 83(13.6%) |  |
| Charlson index ≥ 2, n(%) | | 2,781(55.2%) | 2,467(55.8%) | 314(51.4%) | 0.041 |
