# Supplementary material for: Neutrophil-to-lymphocyte ratio (NLR) predicts mortality in hospitalized geriatric patients independent of the admission diagnosis: a multicenter prospective cohort study
Source: J Transl Med. 2023 Nov 21;21:835. doi: 10.1186/s12967-023-04717-z (PMC10664513; doi:10.1186/s12967-023-04717-z)
Supplement: Supplementary file 2 — Additional file 2: NLR (median and interquartile range) for the main diagnosis at hospital admission. [file 12967_2023_4717_MOESM2_ESM.docx]

**Additional File 2. NLR (median and interquartile range) for the main diagnosis at hospital admission.**

|  | Median (IQR) | Overall *p* |
| --- | --- | --- |
|  |  | <0.001 |
| Diabetes | 7.0 (4.4-11.5) |  |
| Metabolism and nutrition disorders | 6.7 (4.2-11.6) |  |
| Delirium and other psychiatric disorders | 6.0 (3.6-10.6) |  |
| CVD | 3.1 (2.2-5.1) |  |
| Cancer | 4.0 (2.4-7.4) |  |
| Anemia | 5.3 (3.2-10.1) |  |
| Dementia or other disorders of the nervous system | 3.2 (2.2-5.5) |  |
| Heart failure and heart disease | 6.9 (4.4-12.4) |  |
| Hypertension or cardiac arrhythmias | 6.4 (3.6-11.9) |  |
| Lung infections | 8.9 (5.1-14.8) |  |
| Gastrointestinal pathologies | 6.1 (3.7-11.7) |  |
| Genitourinary pathologies | 62 (3.8-11.9) |  |
| Sepsis | 10.0 (5.4-19.1) |  |
| Other | 6.3 (3.7-11.0) |  |
