# Supplementary material for: Neutrophil-to-lymphocyte ratio (NLR) predicts mortality in hospitalized geriatric patients independent of the admission diagnosis: a multicenter prospective cohort study
Source: J Transl Med. 2023 Nov 21;21:835. doi: 10.1186/s12967-023-04717-z (PMC10664513; doi:10.1186/s12967-023-04717-z)
Supplement: Supplementary file 3 — Additional file 3: Comparisons of mean NLR values by main diagnosis at hospital admission (one-way ANOVA and post-Hoc Bonferroni test). [file 12967_2023_4717_MOESM3_ESM.docx]

**Additional File 3. Comparisons of mean NLR values by main diagnosis at hospital admission (one-way ANOVA and post-Hoc Bonferroni test)**

| **Row Mean-**  **Col Mean** | 14. | 1. | 2. | 3. | 4. | 5. | 6. | 7. | 8. | 9. | 10. | 11. | 12. |
| --- | --- | --- | --- | --- | --- | --- | --- | --- | --- | --- | --- | --- | --- |
| 1. | -0.31 |  |  |  |  |  |  |  |  |  |  |  |  |
| 2. | 0.56 | 0.87 |  |  |  |  |  |  |  |  |  |  |  |
| 3. | -1.20 | -0.90 | -1.77 |  |  |  |  |  |  |  |  |  |  |
| 4. | -4.94*** | -4.63 | -5.50*** | -3.74 |  |  |  |  |  |  |  |  |  |
| 5. | -3.38* | -3.07 | -3.94*** | -2.17 | 1.56 |  |  |  |  |  |  |  |  |
| 6. | -1.39 | -1.08 | -1.95 | -0.18 | 3.55*** | 1.99 |  |  |  |  |  |  |  |
| 7. | -4.38*** | -4.08 | -4.95*** | -3.18 | 0.55 | -1.01 | -3.00*** |  |  |  |  |  |  |
| 8. | 0.41 | 0.72 | -0.15 | 1.61 | 5.35*** | 3.79 | 1.80 | 4.79*** |  |  |  |  |  |
| 9. | -0.94 | -0.63 | -1.50 | 0.27 | 4.00*** | 2.44 | 0.45 | 3.45*** | -1.35 |  |  |  |  |
| 10. | 2.85*** | 3.16 | 2.29* | 4.06 | 7.79*** | 6.23*** | 4.24*** | 7.24*** | 2.44 | 3.79*** |  |  |  |
| 11. | 0.34 | 0.65 | -0.22 | 1.54 | 5.28*** | 3.72** | 1.73 | 4.73*** | -0.07 | 1.28 | -2.51*** |  |  |
| 12. | 0.34 | 0.65 | -0.22 | 1.55 | 5.28*** | 3.72** | 1.73 | 4.73*** | -0.07 | 1.28 | -2.51*** | 0.00 |  |
| 13. | 5.17*** | 5.47* | 4.60*** | 6.37*** | 10.11*** | 8.55*** | 6.56*** | 9.55*** | 4.76*** | 6.10*** | 2.32 | 4.83*** | 4.83*** |

Note: 1. Diabetes; 2. Metabolism and nutrition disorders; 3. Delirium and other psychiatric disorders; 4. Cerebrovascular disease; 5. Cancer; 6. Anemia; 7. Dementia or other disorders of the nervous system; 8. Heart failure and heart disease; 9. Hypertension or cardiac arrhythmias; 10. Lung infections; 11. Gastrointestinal pathologies; 12. Genitourinary pathologies; 13. Sepsis; 14. Other

**p <0.05; **: p<0.01; ***: p<0.001.*
