# Supplementary material for: Neutrophil-to-lymphocyte ratio (NLR) predicts mortality in hospitalized geriatric patients independent of the admission diagnosis: a multicenter prospective cohort study
Source: J Transl Med. 2023 Nov 21;21:835. doi: 10.1186/s12967-023-04717-z (PMC10664513; doi:10.1186/s12967-023-04717-z)
Supplement: Supplementary file 4 — Additional file 4: Assessment of the effect modification of diseases and lab values on the association between NLR (both continuous and categorical) and death. [file 12967_2023_4717_MOESM4_ESM.docx]

**Additional File 4: Assessment of the effect modification of diseases and lab values on the association between NLR (both continuous and categorical) and death**

|  | **Continuous NLR** |  | **NLR≥7.95** |  |
| --- | --- | --- | --- | --- |
|  | **Age and sex-adjusted HR (95%CI)** | **AUC** | **Age and sex-adjusted HR (95%CI)** | **AUC** |
| **Sex** |  |  |  |  |
| Male (n=2,141) | 1.03 (1.02-1.03) | 0.6911 | 2.58 (2.00-3.34) | 0.6432 |
| Female (n=2,893) | 1.03 (1.02-1.03) | 0.7210 | 2.94 (2.36-3.67) | 0.6612 |
|  |  |  |  |  |
| **Main diagnosis** |  |  |  |  |
|  |  |  |  |  |
| Sepsis (n=235) | 1.01 (1.00-1.02) | 0.5905 | 1.70 (1.01-2.87) | 0.5723 |
| Main diagnoses other than sepsis (n=4,799) | 1.03 (1.02-1.03) | 0.7091 | 2.76 (2.30-3.31) | 0.6511 |
|  |  |  |  |  |
| Pneumonia (n=787) | 1.02 (1.02-1.03) | 0.6742 | 2.21 (1.50-3.27) | 0.6143 |
| Main diagnoses other than pneumonia (n=4,247) | 1.03 (1.02-1.03) | 0.7080 | 2.78 (2.30-3.37) | 0.6646 |
|  |  |  |  |  |
| Malnutrition (n=369) | 1.02 (1.01-1.03) | 0.7020 | 3.43 (2.05-5.75) | 0.6517 |
| Main diagnoses other than malnutrition (n=4,665) | 1.03 (1.02-1.03) | 0.7048 | 2.65 (2.21-3.18) | 0.6512 |
|  |  |  |  |  |
| Cancer (n=188) | 1.05 (1.00-1.11) | 0.6952 | 2.75 (1.14-6.61) | 0.6810 |
| Main diagnoses other than cancer (n=4,846) | 1.03 (1.02-1.03) | 0.7067 | 2.73 (2.29-3.26) | 0.6500 |
|  |  |  |  |  |
| Anemia (n=410) | 1.03 (1.000-1.06) | 0.6944 | 2.04 (0.88-4.73) | 0.5942 |
| Main diagnoses other than anemia (n=4,624) | 1.03 (1.02-1.03) | 0.7049 | 2.70 (2.27-3.22) | 0.6576 |
|  |  |  |  |  |
| Cerebrovascular disease (n=259) | 1.08 (1.02-1.13) | 0.7469 | 3.40 (0.87-13.26) | 0.6771 |
| Main diagnoses other than disease (n=4,775) | 1.03 (1.02-1.03) | 0.6976 | 2.62 (2.21-3.11) | 0.6567 |
|  |  |  |  |  |
| Congestive heart failure (n=151) | 1.02 (0.99-1.04) | 0.5695 | 1.63 (0.81-3.29) | 0.5586 |
| Not congestive heart failure (n=4,883) | 1.03 (1.02-1.03) | 0.7115 | 2.80 (2.35- 3.35) | 0.6612 |
|  |  |  |  |  |
| **Renal function, hemoglobin values and nutritional status** |  |  |  |  |
|  |  |  |  |  |
| eGFR > 60 (n=1,173) | 1.05 (1.03-1.06) | 0.7458 | 3.33 (2.18-5.09) | 0.6817 |
| eGFR < 60 (n=3,861) | 1.03 (1.02-1.03) | 0.6905 | 2.64 (2.20-3.17) | 0.6411 |
|  |  |  |  |  |
| eGFR < 45 (n=2,487) | 1.02 (1.02-1.03) | 0.6583 | 2.27 (1.86-2.77) | 0.6175 |
| eGFR > 45 (n=2,547) | 1.03 (1.03-1.04) | 0.7441 | 3.87 (2.84-5.27) | 0.6991 |
|  |  |  |  |  |
| Hypoalbuminemia (n=2,847) | 1.02 (1.02-1.03) | 0.6451 | 2.14 (1.77-2.59) | 0.6023 |
| Normal albuminemia (n=2,187) | 1.05 (1.04-1.07) | 0.7078 | 2.86 (1.79-4.57) | 0.6621 |
|  |  |  |  |  |
| Hemoglobin < 12 g/dL in women or < 13 g/dL in men (n=3,503) | 1.03 (1.02-1.03) | 0.6956 | 2.72 (2.25-3.29) | 0.6522 |
| Normal hemoglobin (n=1,531) | 1.03 (1.02-1.03) | 0.7355 | 2.88 (2.01-4.12) | 0.6810 |
|  |  |  |  |  |
| Patients with eGFR > 45, absence of sepsis, and normal hemoglobin (n=917) | 1.02 (1.00-1.05) | 0.7646 | 4.30 (2.19-8.45) | 0.7255 |
