# Supplementary figures and images for: Neutrophil-to-lymphocyte ratio (NLR) predicts mortality in hospitalized geriatric patients independent of the admission diagnosis: a multicenter prospective cohort study
Source: J Transl Med. 2023 Nov 21;21:835. doi: 10.1186/s12967-023-04717-z (PMC10664513; doi:10.1186/s12967-023-04717-z)

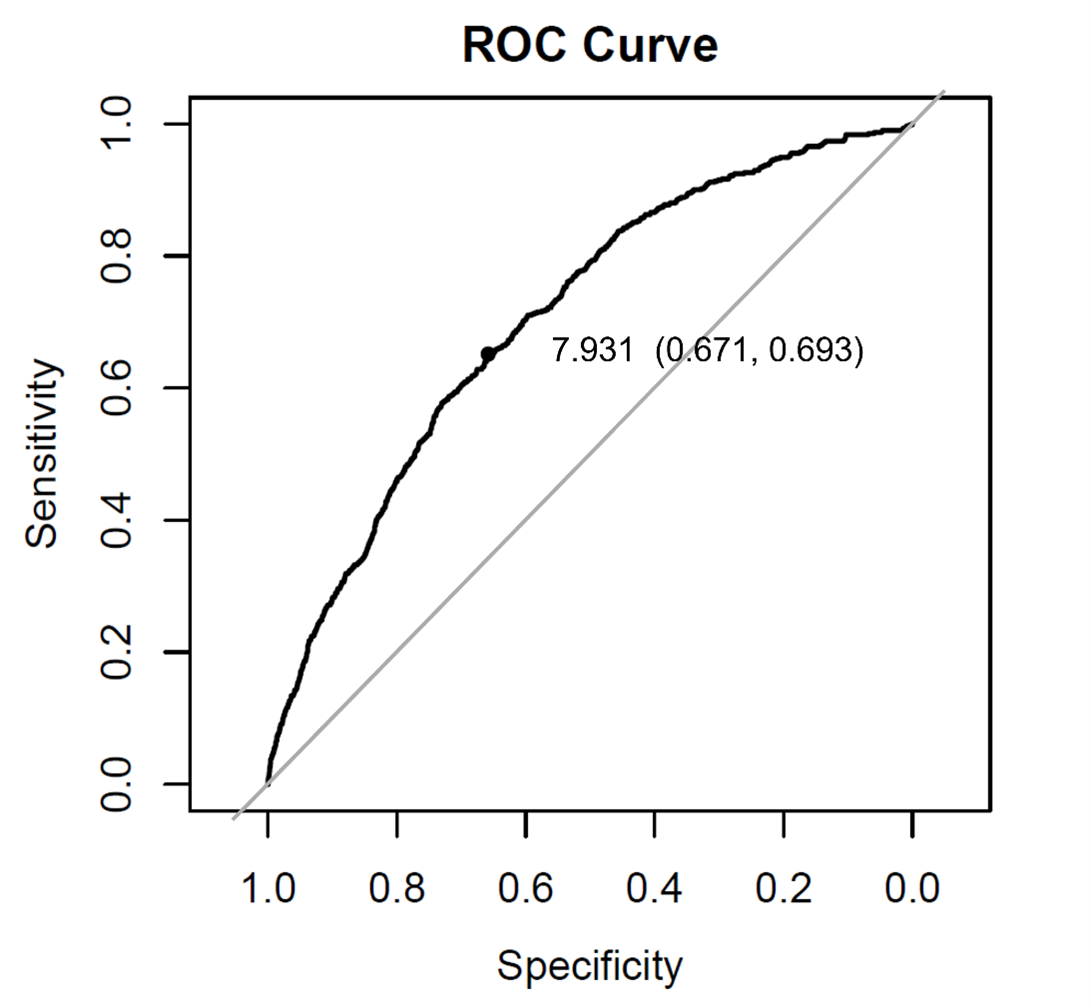

Supplement: Supplementary file 5 — Additional file 5: Figure S1a. Optimal NLR cut-off for increased risk of in-hospital mortality in men and women. Selection of the optimal cut-off of NLR for increased risk of in-hospital mortality by using sex-weighted and age-adjusted ROC curve in the study population. [file 12967_2023_4717_MOESM5_ESM.tif]

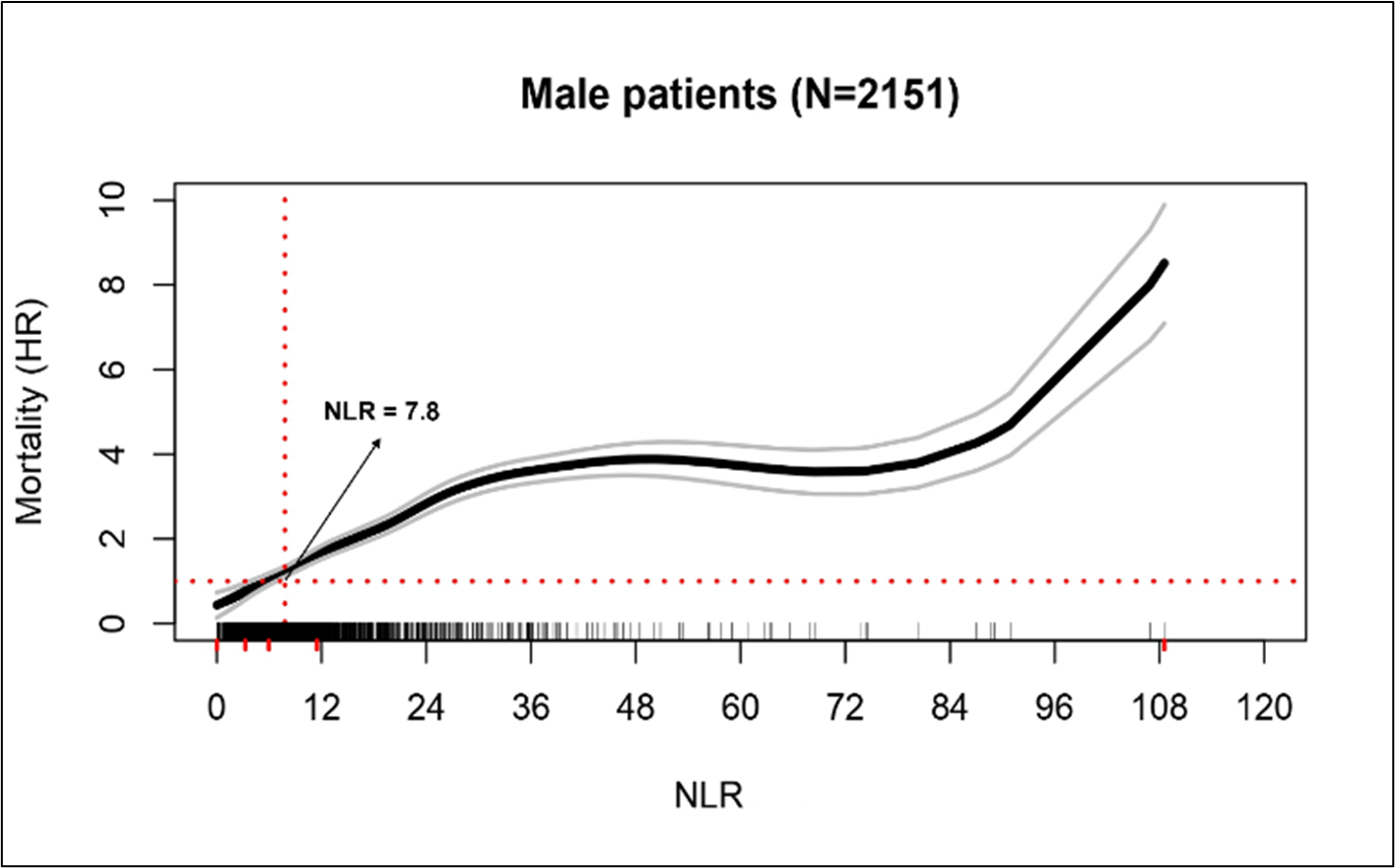

Supplement: Supplementary file 6 — Additional file 6: Figure S1b. Restricted cubic spline showing the non-linear relationship between NLR and in-hospital mortality among male patients. Red ticks on x-axis represent quartiles of NLR distribution. [file 12967_2023_4717_MOESM6_ESM.tif]

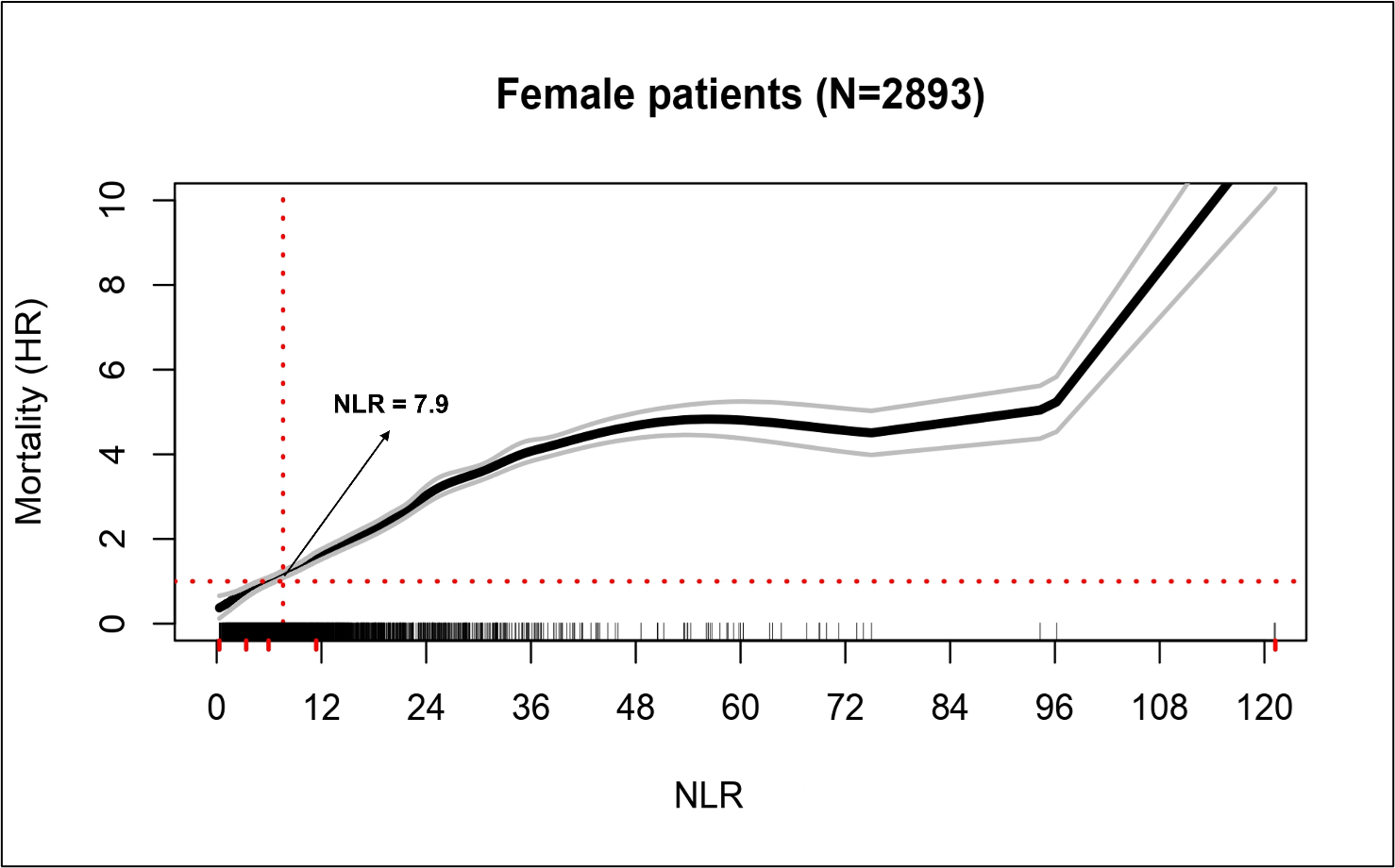

Supplement: Supplementary file 7 — Additional file 7: Figure S1c. Restricted cubic spline showing the non-linear relationship between NLR and in-hospital mortality among female patients. Red ticks on x-axis represent quartiles of NLR distribution. [file 12967_2023_4717_MOESM7_ESM.tif]

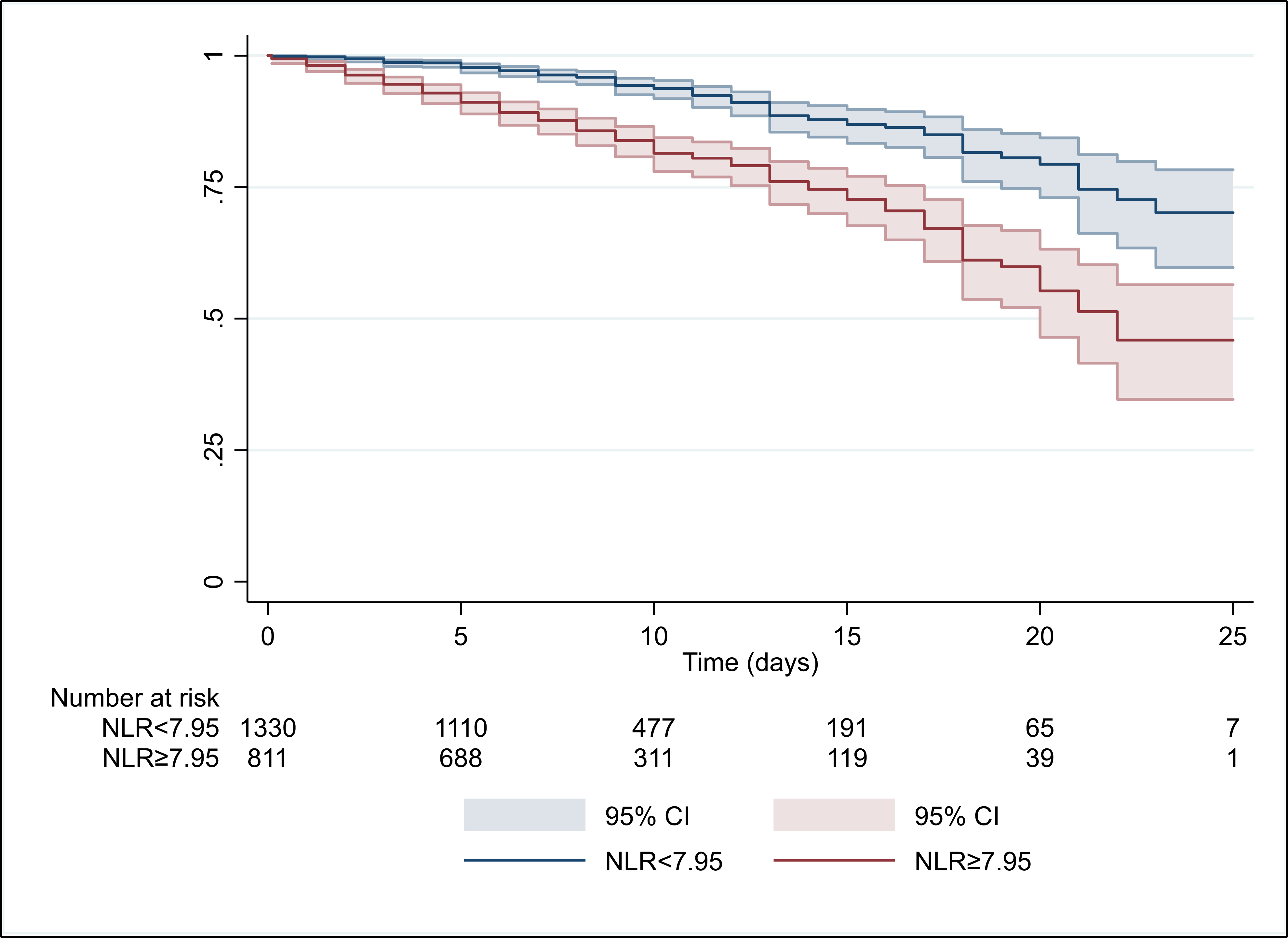

Supplement: Supplementary file 8 — Additional file 8: Figure s2a. Kaplan Meier curves showing survival of male patients according to NLR categories. [file 12967_2023_4717_MOESM8_ESM.tif]

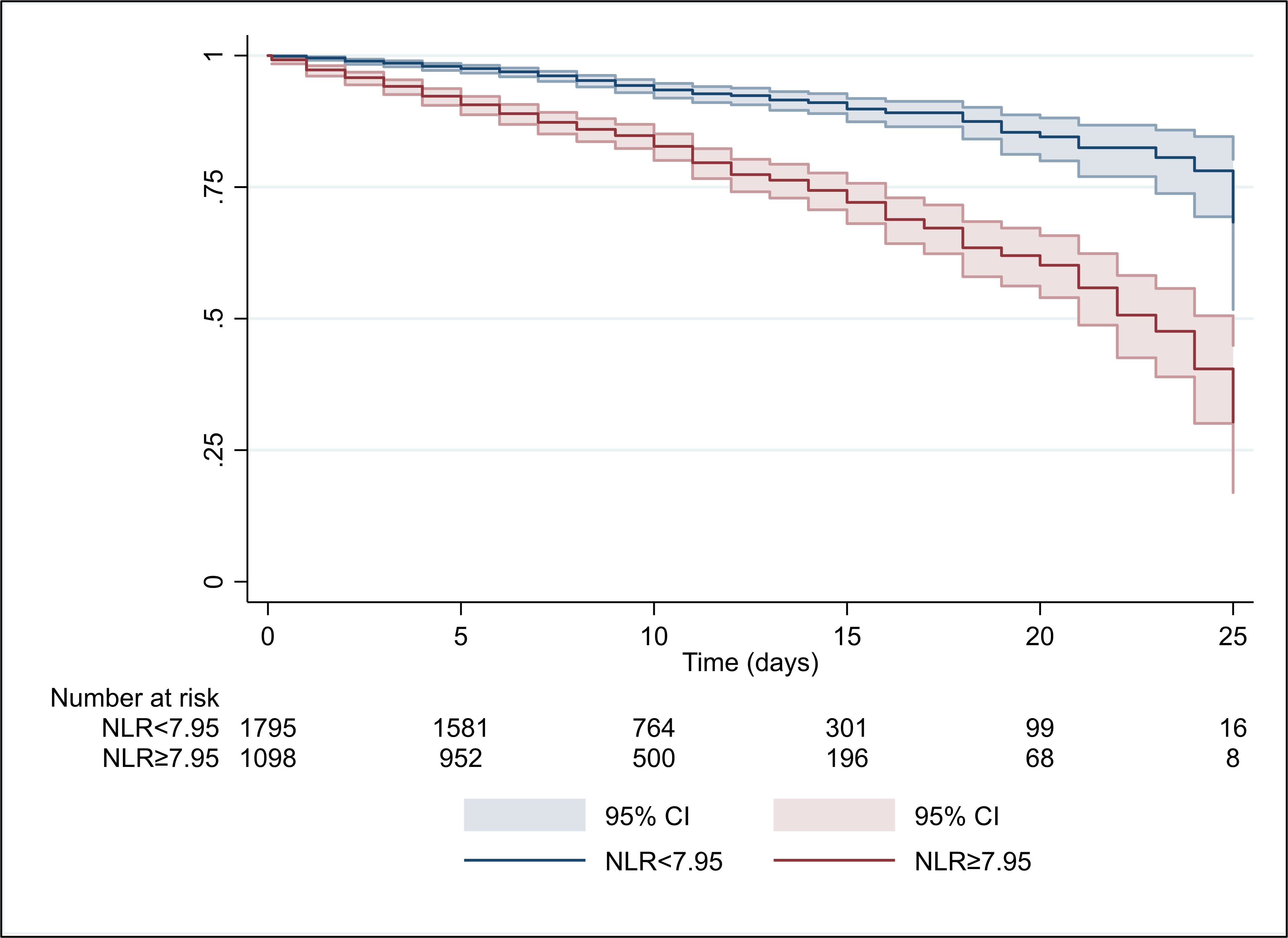

Supplement: Supplementary file 9 — Additional file 9: Figure s2b. Kaplan Meier curves showing survival of female patients according to NLR categories. [file 12967_2023_4717_MOESM9_ESM.tif]
